# Semi-quantitative CT imaging in improving visualization of faint ground glass opacities seen in early/mild coronavirus (covid-19) cases

**DOI:** 10.1186/s43055-020-00354-4

**Published:** 2020-12-01

**Authors:** Rasha Mostafa Mohamed Ali, Mai Bahgat Ibrahim Ghonimy

**Affiliations:** 1 Radiology Department, Kasr Alaini Hospitals Cairo University, Cairo, Egypt; 2 Diagnostic & Interventional Radiology Department, Thoracic and Gastrointestinal Tract Imaging Units, Kasr Al-Aini Hospitals, Faculty of Medicine Cairo University, Cairo, Egypt

**Keywords:** Semi-quantitative; CT imaging; faint GGO; early /mild COVID-19

## Abstract

**Background:**

Chest CT is an essential and simple diagnostic method for early detection of pulmonary changes in COVID-19 patients. Semi-quantitative technique depending on both visual and color coded images helps to improve the early detection of COVID-19 chest affection and thus help to control spread of infection.

**Results:**

From first of May to July 15, 2020, 30 patients in Cairo, Egypt who have positive RT-PCR tests and positive pulmonary manifestation were included in our study, 26 patients (86.6%) with faint ground glass opacities were detected by both visual and color coded images, while in 4 patients (13.3%) were only visualized by color coded images and confirmed by CT density assessment.

**Conclusion:**

The combined use of visual and color coded images enhance and improve the early detection of faint ground glass opacities seen in early COVID-19 affection.

## Background

Chest CT plays a vital role in the assessment and follow up for patients with positive COVID-19 infection [1].

Radiological examinations are vital in early diagnosis and assessment of disease course, as most COVID-19 infected patients were diagnosed with characteristic CT imaging patterns [1].

In absence of specific therapeutic drugs or vaccines for 2019 novel coronavirus disease (COVID-19), it is essential to detect the diseases at an early stage, and immediately isolate the infected person from the healthy population [2].

Chest CT, as a routine imaging tool for pneumonia diagnosis, is relatively easy to perform and can produce fast diagnosis [3].

The chest CT scans showed a higher sensitivity for the diagnosis of COVID-19 infection than initial RT-PCR results [2].

Thin-slice chest CT plays a vital role in early detection, observation, and disease evaluation [4].

The aim of this study is to assess the significance of color coded images in the enhancement of visualization of faint ground glass opacities that were the only manifestation in early affected COVID-19 patients.

## Methods

### Ethical consideration

All patients provided written informed consent. The results of this research were used only in scientific purposes and not in any other aims.

Ethical approval wasn’t applicable when we started our research in May 2020 due to current situation of coronavirus crisis.

### Study Design

This prospective study included 30 patients (21 males, 9 females) with age range from 25 to 65 years (mean age of 34.2 years) confirmed to be infected with SARS-CoV-2, referred for MSCT assessment of the chest (Table 1). MSCT of the chest was done to all patients as requested. The study was conducted between first of May and July 12, 2020, in Cairo, Egypt.

**Table 1:** MDST technique

Tube voltage	120 kVp
Tube current	60 – 120 mAp
Slice thickness	1 mm
Reconstruction interval	1 mm
Patient position	Supine
Respiration	Breath hold full inspiration
Matrix size	512 × 512

### Inclusion criteria

Laboratory proven PCR positive COVID-19 patients with faint ground glass opacities in MSCT chest.

### Exclusion criteria

Patient’s CT showing consolidative patches.

Patients who recently experienced clinically defined pulmonary infection attributable to other pathogens.

Patients with severe artifacts on CT images.

### Methods

All enrolled patients were subjected to:

- ❖ Through history taking.
- ❖ Laboratory assessment (patients with positive PCR test).
  • Computed tomography (CT) of the chest:
  • CT scan chest was done to all patients using 64 channels MSCT.
  - The detailed parameters for CT acquisition were as follows:
- Tube voltage, 120–160 kVp.
- tube current, standard (reference mAs, 60–120)
- Slice thickness, 1.0 mm.
- Reconstruction interval, 1.0–3.0 mm
- Using a sharp reconstruction algorithm.
- CT images were obtained with the patient in the supine position with suspended full inspiration and without contrast medium (Table 1)
- All images were viewed on both lung (width, 1500 HU; level, − 700 HU) and mediastinal (width, 350 HU; level, 40 HU) settings.
  • VR (3 colors, 3D volume rendering lung images):
- The images acquired were sent to a separate workstation and using certain DICOM viewer to be processed, manipulated.
- Threshold limits of -500 to -1024 HU were applied to exclude soft tissue surrounding the lung and large vessels within the lung
- Selecting lung density analysis preset, the lungs are automatically segmented from chest wall, mediastinum, airways and vessels.
- This software and Dicom viewer automatically analyses the density distribution of the lungs into different colors by using color mask tool, by entering the density range, selecting the desired color, which indicates the area that the HU ranges occupy in the image.
- Normally aerated (%NAL, − 501, − 900 HU) and the value range of − 750 HU to − 300 HU and − 300 HU to 50 HU was defined as GGO and consolidation, respectively [5]
  • The chest CT scan was evaluated by two expert radiologist separately searching for the faint ground glass opacities in axial lung window images as well as color coded images**.**
  • Semi-quantitative method depends on measuring HU unit of the lesion as well as it relays on the color coded image assessment while the quantitative method is an objective method that depend only upon HU values calculation.

### Statistical analysis

Owing to small sample size, findings are presented as medians and interquartile ranges.

## Results

This cross section study included 30 patients (21 males, 9 females) with age range from 25 to 65 years (mean age of 34.2 years) confirmed to be infected with SARS-CoV-2 using RT-PCR test. MSCT of the chest was performed to all patients and processed in a separate work station and certain DICOM viewer.

All patients had history of direct or indirect contact with COVID-19 infected people. The interval from onset symptoms to first chest CT scan was 6–8 days. The common first symptoms were fever seen in 23 patients (77.4%), dry cough in 18 patients (60%), while 10 patients (33.3%) presented with dyspnea.

The most imminent radiological finding was ground glass opacity seen in 26 patients (86.6%) were detected by both visual and color coded images, while in 4 patients (13.3%) the GGO were very faint and were only visualized by color coded images and confirmed by CT density assessment.

The size of the lesions ranged in diameter from 1 to 3 cm. 24 patients showed lower zone predominance ( 80%), 4 patients showed equal distribution between the upper and lower zones ( 13.3%) and two patients showed upper zone predominant changes ( 6.6%) (Table 2).

**Table 2:** Ground glass distribution

Percent	Number of cases	Predominant distribution
80%	24	lower lobe predominance
6.6%	2	Upper lobe predominance
13.3%	4	Equal distribution in upper and lower lobe
93.3%	28	Peripheral distribution
6.6%	2	Peripheral and perihilar distribution

The faint ground-glass opacities were peripheral in most patients 28 (93.3%), while 2 patients showed both peripheral & peri-hilar distribution (6.6%) (Table 2).

## Discussion

The crisis of Coronavirus disease 2019 (COVID-19) has recently attracted the attention all over the world. (COVID-19), is a highly infectious disease caused by severe acute respiratory syndrome coronavirus 2 (SARS-CoV-2) [6].

Currently, there are no validated anti-viral medications or other specific therapies targeted toward the treatment of COVID-19. Management of this disease is symptomatic and, more importantly, focused on public health measures which slow the spread of disease, an epidemiological concept referred to as “flattening the curve” [7].

Therefore, early recognition of patients with COVID-19 (radiologic or otherwise) is critical in order to isolate these cases and prevent additional infection [8].

Computed tomography (CT) is an important and essential method for the diagnosis and evaluation of the severity of COVID-19 as well as monitoring disease progression and evaluating the therapeutic efficiency [9].

**Fig. 1:**
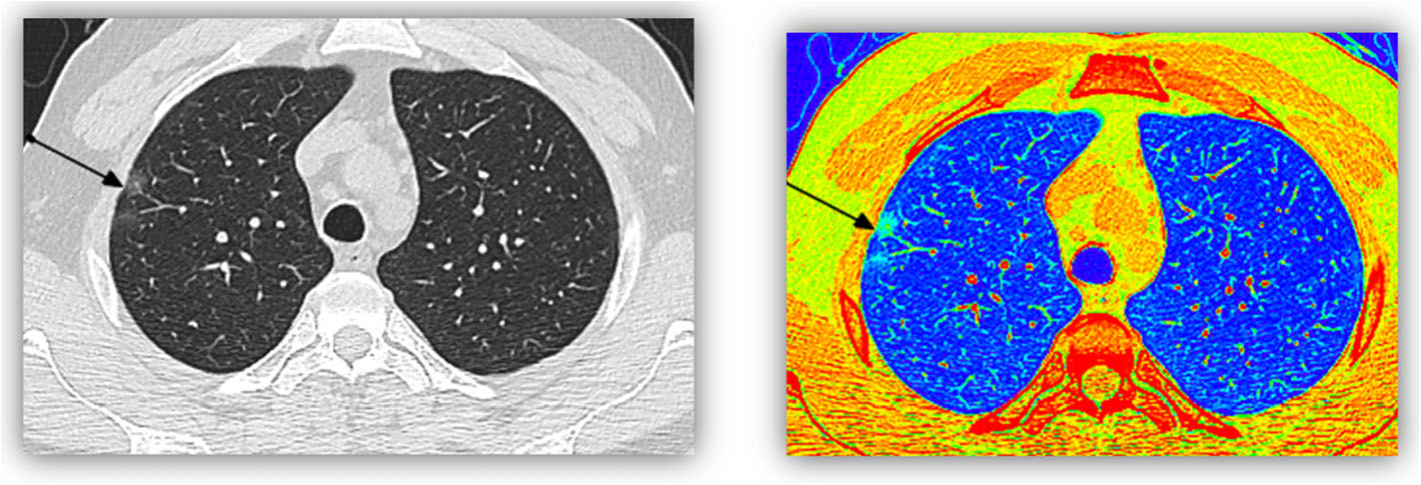
A male patient 32 years old, presented with mild dyspnea and COVID-19 positive PCR. MDCT was done with color coded images, MDCT showed right upper lobe tiny ground glass pulmonary nodule (2 mm) which was confirmed in color coded image (as a green nodule whose HU above -750 HU and normal lung appear blue color -750 to -950 HU)

Quantitative imaging analysis (QIA), which allows for precise identification of lung tissue density by Hounsfield units (HU), can help differentiate otherwise subtle radiographic diagnoses (Fig. 1) [10].

This cross section study included 30 patients (21 males, 9 females) with male predominance 70%. Age range from 25 to 65 years (mean age of 34.2 years). All patients confirmed to be infected with SARS-CoV-2 using RT-PCR test.

MSCT of the chest was done to all patients as requested & the acquired images were sent to a separate workstation using certain DICOM viewer to be processed & manipulated.

The study was conducted between first of May and July 12, 2020, in Cairo, Egypt.

The interval from onset symptoms to first chest CT scan was 6–8 days. The common first symptoms were fever seen in 23 patients ( 77.4%), dry cough in 18 patients ( 60%), while 10 patients ( 33.3%) presented with dyspnea which agrees with study done by Dong Sun, et al. [11].

In our study we noted, ground-glass opacity (GGO) is the main CT findings in patients with mild/early COVID-19 and was seen in 26 patients (86.6%) & it was essentially associated with the course and severity of the disease which agrees with the study done by and Feng Z et al. [12].

**Fig. 2:**
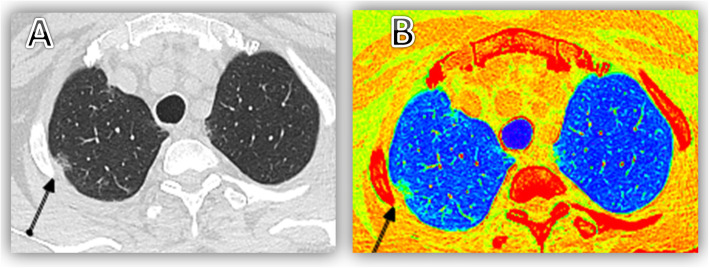
56 years old male patient presented with fever & chest symptoms including dry cough, yet CT chest was unremarkable (**a**) except for right upper lobe apical segment subpleural ill-defined faint small ground glass opacity, Which became more obvious in color coded images (**b**) (green in color)

The detection and recognition of GGO is based on a subjective assessment of lung attenuation at CT, therefore, CT should be performed within objective parameters that make lesion depiction reliable and reproducible (Fig. 2) [13].

**Fig. 3:**
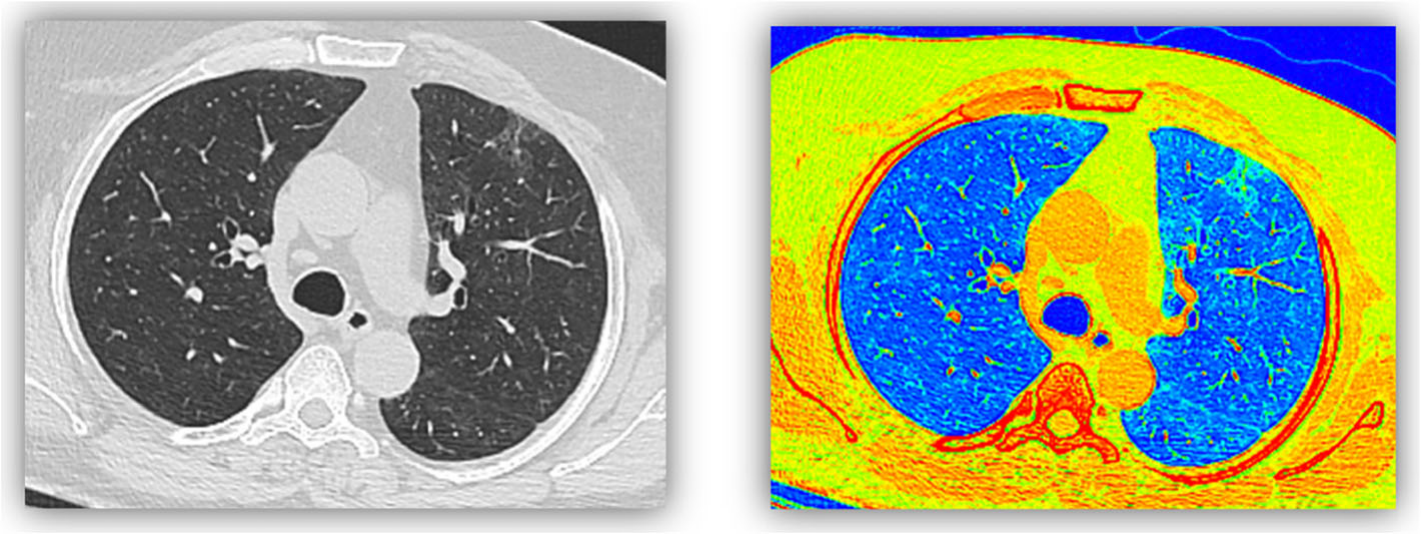
34 years old male patient who had mild fever, with postive PCR. MDCT was done showing left upper lobar anterior segment suspected faint subpleural opacity, which was much prominent in color coded images (as small green patch)

GGOs may not always be obvious on CT images, and they may be missed. The recognition of GGO is based on a subjective assessment of lung attenuation at CT (Fig. 3) [14].

**Fig. 4:**
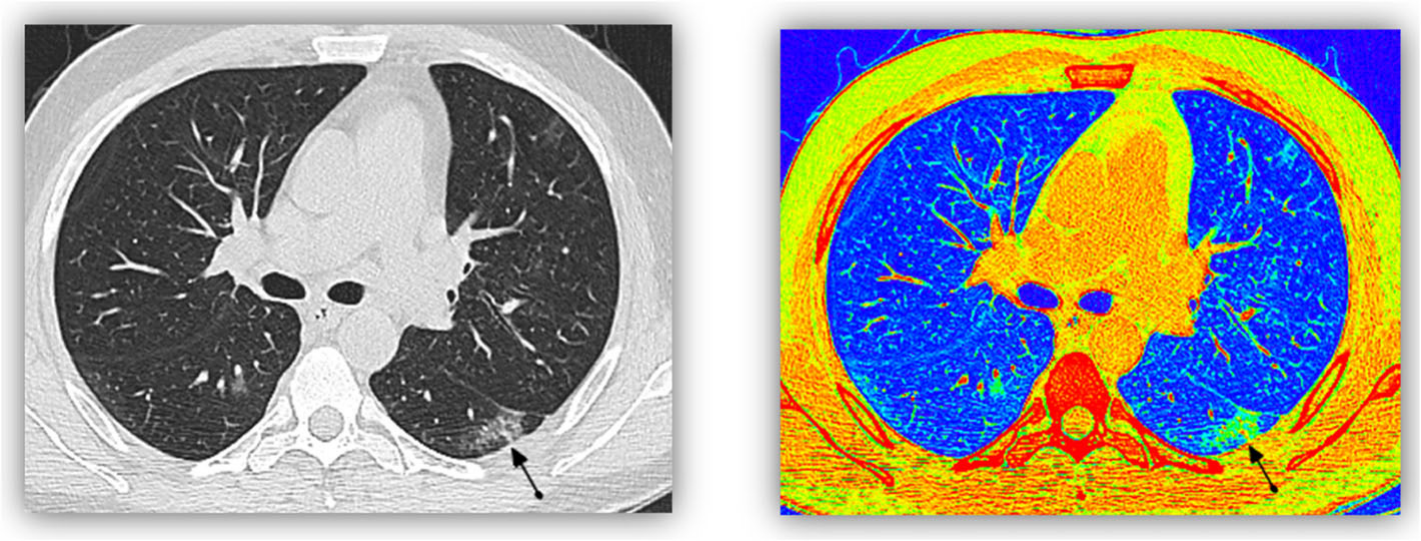
22 years old male patient with high grade fever and cough. MDCT was done showing left lower lobe apical segment subpleural patchy area of ground glass opacity. On doing additional color coded images two additional small nodular opacities became more clear in the right lower lobe apical segment and left upper lobe

In the present study, ground glass opacity seen in 26 patients (86.6%) were detected by both visual and color coded images, while in 4 patients (13.3%) the GGO were very faint and were only visualized by color coded images and confirmed by CT density assessment (Fig. 4).

The size of the lesions ranged in diameter from 1 cm–3 cm. and that goes with study of Dong Sun, et al. that showed correlation between size of lesion and severity of symptoms [11].

**Fig. 5:**
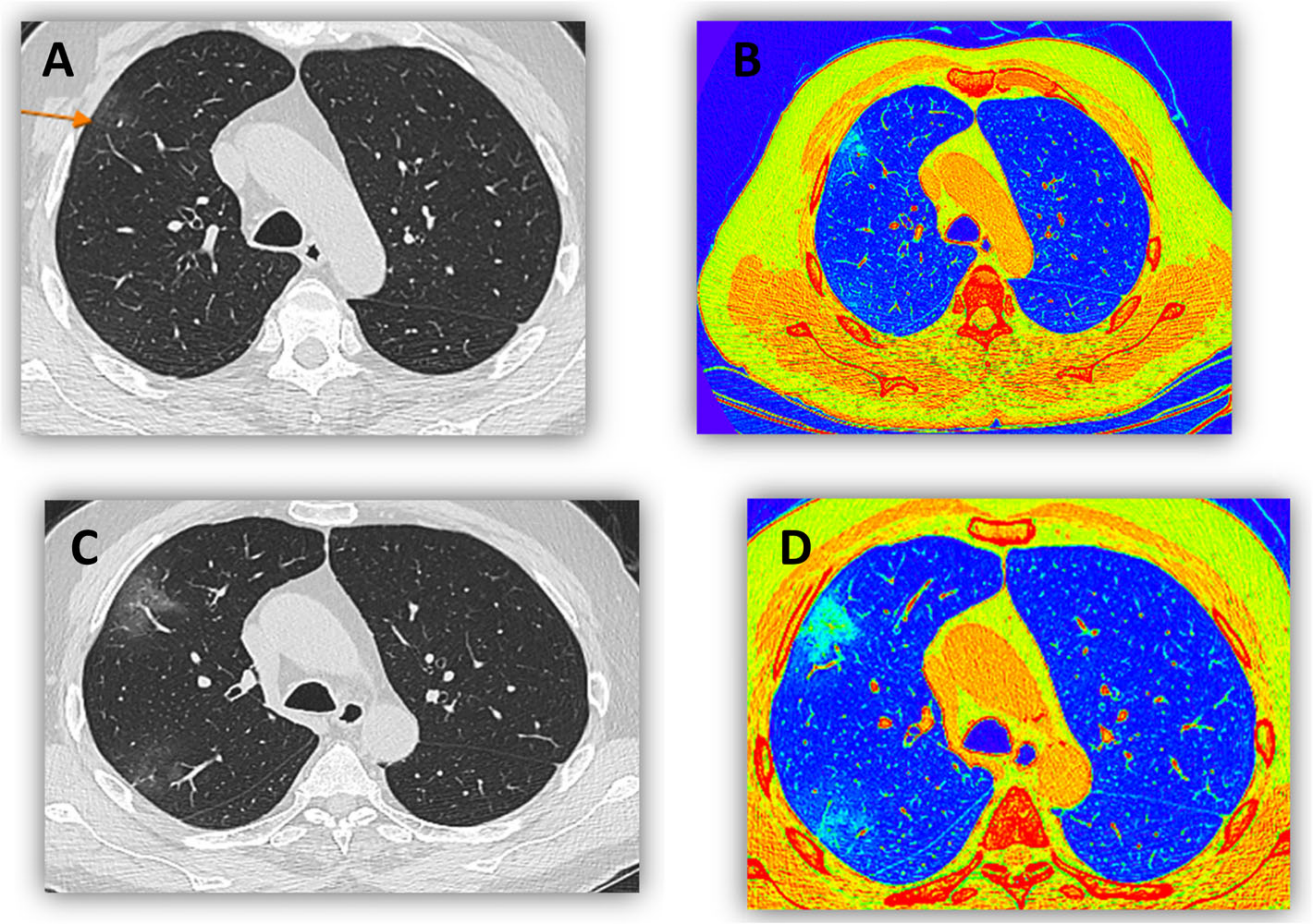
A male patient 42 years old, presented with dull aching chest pain with PCR postive tests. First MDCT (image **a**) was done, showing suspected faint ground glass opacity, additional color coded images (**b**) showed a right upper lobar apical segment area of abnoraml green color (HU more than -750). Follow up CT done after 8 days ( C& D) showed bilateral scattered mainly peripheral ground glass opacities

The early identification of patients and assessment of the severity of COVID-19 may guide clinical treatment options and reduce the mortality rate. In the present study, a using both visual and color coded images helped in confirming diagnosis of mild/early cases of COVID-19 and that was confirmed by study done by Dong Sun,et al. (Fig. 5) [11].

In summary, this study revealed that the combined use of visual and color coded images enhance and improve the early detection of faint ground glass opacities seen in early COVID-19 affection which could help in improving the disease prognosis as well as limiting spread of this highly contagious disease.

## Conclusion

In absence of specific therapeutic drugs or vaccines for (COVID-19), the early identification of COVID-19 is of great value as it may guide clinical treatment options and reduce the mortality rate.

GGO diagnosis remains a diagnostic challenge, Although CT represents a fundamental diagnostic tool because of its sensitivity, and it still needs to be integrated with clinical data to achieve the best clinical management.

The combined use of visual and color coded images enhance and improve the early detection of faint ground glass opacities seen in early COVID-19 affection which could help in improving the disease prognosis as well as limiting spread of this highly contagious disease.

## Data Availability

Data available within the article or its supplementary materials.

## Abbreviations

2019-nCOV: 2019 Novel coronavirus
ARDS: Acute respiratory distress syndrome
COVID-19: Corona Virus Disease
CT: Computed Tomography
DICOM: Digital Imaging and Communications in Medicine
GGO: Ground glass opacity
HU: House field Unit
IV: Intra-Venous
MDCT: Multi-Detector Computed Tomography
MSCT: Multi-slice Computed Tomography
NAL: Normally aerated lung
RT-PCR: Reverse transcriptase polymerase chain reaction
PCR: Polymerase chain reaction
SARS-cov-2: Severe acute respiratory syndrome coronavirus 2
